# Structure of the cobalamin-binding protein of a putative *O*-demethylase from *Desulfitobacterium hafniense* DCB-2

**DOI:** 10.1107/S0907444913011323

**Published:** 2013-07-20

**Authors:** Hanno Sjuts, Mark S. Dunstan, Karl Fisher, David Leys

**Affiliations:** a Manchester Institute of Biotechnology, Faculty of Life Sciences, University of Manchester, 131 Princess Street, Manchester M1 7DN, England

**Keywords:** *O*-demethylation, cobalamin-binding proteins, *Desulfitobacterium hafniense*

## Abstract

This study describes the identification and the structural and spectroscopic analysis of a cobalamin-binding protein (termed CobDH) implicated in *O*-demethylation by the organo­halide-respiring bacterium *Desulfitobacterium hafniense* DCB-2. The 1.5 Å resolution crystal structure of CobDH is presented in the cobalamin-bound state and reveals that the protein is composed of an N-terminal helix-bundle domain and a C-­terminal Rossmann-fold domain, with the cobalamin coordinated in the base-off/His-on conformation similar to other cobalamin-binding domains that catalyse methyl-transfer reactions. EPR spectroscopy of CobDH confirms cobalamin binding and reveals the presence of a cob(III)alamin superoxide, indicating binding of oxygen to the fully oxidized cofactor. These data provide the first structural insights into the methyltransferase reactions that occur during *O*-demethylation by *D. hafniense*.

## Abstract 2

The first crystal structure of the vitamin B12-binding protein from a three-component *O*-demethylase enzyme system is reported. During *O*-demethylation methyl groups are transferred from phenyl methyl ethers to tetrahydrofolate *via* methyl-B12 intermediates.

## 1. Introduction

Lignin breakdown by fungal haloperoxidases in forest soil can give rise to chlorinated phenyl methyl ethers. Such compounds can serve as the terminal electron acceptors for anaerobic organo­halide-respiring bacteria, leading to the corresponding dechlorinated phenyl methyl ethers (Bache & Pfennig, 1981 ▶; Villemur, 2013 ▶). Similar to acetogenic bacteria, certain strains of organohalide-respiring species have been found to use phenyl methyl ethers as an energy source, leading to further mineralization of lignolic compounds. A transcriptional study noted the upregulation of a gene cluster potentially involved in vanillate metabolism in *Desulfitobacterium hafniense* Y-­51 which includes cobalamin-dependent enzymes (Peng *et al.*, 2012 ▶). The recent availability of genome sequences from several organohalide-respiring bacteria has revealed, in addition to a surprising wealth of reductive dehalogenase genes (*RdhA*s), the presence of multiple putative cobalamin-binding proteins (Kim *et al.*, 2012 ▶). These are often located in gene clusters that appear to encode multicomponent *O*-demethylase-like enzyme systems (Studenik *et al.*, 2012 ▶).

Additionally, it has recently been reported that *D. hafniense* strain PCP-1 is able to catalyse both dehalogenation and *O*-­demethylation reactions stepwise from the same substrate precursors (*e.g.* tetrachloroguaiacol, tetrachloroveratrole or pentachloroanisole), providing a functional link between reductive dehalogenation and *O*-demethylation in organo­halide-respiring bacteria (Villemur, 2013 ▶).

*O*-Demethylases belong to the class of cobalamin-dependent methyltransferases that catalyse methyl-group transfer from a methyl donor to a methyl acceptor *via* a methyl­cobalamin or methylcobamide acting as a reaction intermediate. Such methyl-transfer reactions are of fundamental importance in energy generation in many microorganisms (Matthews *et al.*, 2008 ▶).

In the case of the *O*-demethylase system, four different proteins are needed for the reaction: (i) a substrate-binding domain that serves as a methyl donor, (ii) a cobalamin-binding domain that catalyses the methyl-transfer reaction, (iii) a tetrahydrofolate-binding domain that accepts the methyl group, thus forming 5-methyltetrahydrofolate, and (iv) an activating enzyme that reduces the cobalt of cobalamin back to the catalytically active +1 state in case of occasional oxidation to the inactive +2 state (Kaufmann *et al.*, 1998 ▶; Engelmann *et al.*, 2001 ▶).

Cobalamin-dependent isomerases are responsible for the rearrangement of carbon skeletons (Banerjee & Ragsdale, 2003 ▶). Adenosylcobalamin is usually the required cofactor of this class and it is used to generate substrate radicals as a prerequisite for the catalysed reaction (Matthews, 2009 ▶). Less characterized cobalamin-dependent enzymes are the reductive dehalogenases, which have only been found in anaerobic organohalide-respiring bacteria (Wohlfarth & Diekert, 1997 ▶; Futagami *et al.*, 2008 ▶; Hug *et al.*, 2013 ▶). Reductive dehalogenases catalyse the terminal electron-transfer step in this pathway, which leads to dechlorination (Smidt & de Vos, 2004 ▶).

The ability of cobalamin to effectively catalyse this diverse range of reactions can be attributed to its inherent properties. Firstly, the central Co atom can adopt three different oxidation states (+1, +2 and +3) and is thus able to span a wide range of electron potentials. Furthermore, the upper axial ligand of cobalamin can be substituted by several functional groups (methyl, hydroxo, cyano or adenosyl; Kräutler, 2005 ▶) depending on the reaction being performed.

The first crystal structure of a cobalamin-binding protein to be solved was that of the *Escherichia coli* methionine synthase (MetH), which catalyses the formation of methionine from homocysteine by a methyl-group transfer *via* methyl­cobalamin (Drennan *et al.*, 1994 ▶). More recently, the crystal structure of MtaC, an enzyme that transfers a methyl group from methanol to coenzyme M, was solved (Hagemeier *et al.*, 2006 ▶). In both cases the cobalamin-binding domain adopts a Rossmann fold and the cofactor is bound in a base-off/His-on conformation. Structures of adenosyl-cobalamin isomerases have also been solved, the first of which, the methylmalonyl-coenzyme A mutase, revealed that the cobalamin cofactor likewise binds to a Rossmann-fold domain that positions the cobalamin into a substrate-binding triosephosphate isomerase (TIM) barrel domain (Mancia *et al.*, 1996 ▶). No structural information is currently available for any of the reductive dehalogenases.

Here, we provide the first crystal structure of a previously uncharacterized cobalamin-binding protein (termed CobDH) from *D. hafniense* DCB-2, which is implied to be a central part of the *O*-demethylation enzyme system based on its operon content. CobDH was crystallized in the presence of methylcobalamin and the structure was refined to 1.5 Å resolution. Despite its relatively low sequence homology (28%), the crystal structure of CobDH revealed significant structural homology to previously determined methyltransferase structures. The structural data are corroborated by EPR and UV–­Vis spectroscopy and show that the cobalamin bound to CobDH can populate the three oxidation states +1, +2 and +3.

## 2. Materials and methods

### 2.1. Cloning, expression and purification

The *Dhaf0720* gene was synthesized with codon optimization for expression in *E. coli* by the MWG operon. PCR amplification was performed with the primers 5′-AAGTTC­TGTTTCAGGGCCCGGTCATCGACCTGAATGCG-3′ and 5′-ATGGTCTAGAAAGCTTTAACCTACCCAACGTTGGC-3′ containing 5′ overhangs that are compatible with ligation-independent cloning (TaKaRa Bio Inc.). The gene was inserted into the pOPINF vector (OPPF, UK) encoding an N-­terminal hexahistidine tag and an HRV 3C recognition sequence (LEVLFQ/GP) using the restriction sites *Hin*dIII and *Kpn*I.

*E. coli* BL21 (DE3) cells (Merck) were transformed with the generated plasmid. The cells were grown in LB medium at 310 K and shaken until they reached mid-log phase. At this point the temperature was lowered to 295 K and protein expression was induced by the addition of IPTG (1 m*M* final concentration). Cells were harvested after 12–16 h by centrifugation; they were resuspended in buffer *A* [50 m*M* HEPES pH 8, 500 m*M* NaCl, 5%(*v*/*v*) glycerol, 30 m*M* imidazole] supplemented with DNase, RNase, lysozyme (Sigma) and Complete EDTA-free protease inhibitor (Roche) and stirred on ice for 30 min. Methylcobalamin (Sigma) was added to the cells before they were lysed by sonication (Bandelin) and insoluble cell membranes were removed by ultracentrifugation at 98 000*g* for 45 min at 277 K. The soluble crude extract was loaded onto a 5 ml Ni–NTA column equilibrated with buffer *A*. After loading, the column was washed with ten column volumes of buffer *A* in order to remove nonspecifically bound protein. CobDH was eluted using a linear gradient to buffer *B* (buffer *A* plus 270 m*M* imidazole). Fractions containing CobDH were pooled, concentrated to 10 ml and then diluted to 100 ml with 50 m*M* HEPES pH 8. The sample was then directly loaded onto a 20 ml Mono Q anion-exchange column and eluted with a linear gradient of 0–500 m*M* NaCl in 50 m*M* HEPES pH 8. To cleave off the His tag, fractions containing the target protein were pooled, concentrated and incubated for 16–20 h with HRV 3C protease (Novagen) at a 1:100 molar ratio. The cleaved His tag was removed by reverse Ni–NTA affinity chromatography before a final purification step on an S200 gel-filtration column equilibrated with 10 m*M* HEPES pH 8, 100 m*M* NaCl. In order to assure complete cofactor reconstitution, some excess methylcobalamin was added to the sample prior to the gel-filtration step.

### 2.2. Crystallization and cryoprotection

Holo CobDH was concentrated to ∼15 mg ml^−1^ in 10 m*M* HEPES pH 8, 100 m*M* NaCl using a 10 kDa molecular-weight cutoff spin concentrator (Sartorius). Crystallization screens were performed by the sitting-drop vapour-diffusion method and drops were dispensed (200 nl protein solution plus 200 nl mother liquor equilibrated against 50 µl mother liquor in the reservoir) in a 96-well format using a high-throughput liquid-handling robot (Mosquito MD11-11, Molecular Dimensions). Small needle-shaped crystals grew from 0.01 *M* zinc chloride, 0.1 *M* HEPES pH 7, 20%(*w*/*v*) PEG 6000 within 7 d. The entire drop containing these small crystals was resuspended in 40 µl of the mother liquor from the reservoir. The crystals were crushed in this solution by the addition of a microseed bead (Molecular Dimensions) and rigorous vortexing for 2 min. The thus generated microcrystals were used without further dilution as nucleation seeds in a second round of screening using the sitting-drop vapour-diffusion method and the PACT premier matrix screen (Molecular Dimensions) as described by D’Arcy *et al.* (2007 ▶). Seed drops consisted of 200 nl purified CobDH (in the same buffer and at the same concentration as used for the initial crystallization screen) plus 100 nl nucleation seed plus 300 nl mother liquor and were equilibrated against 50 µl mother liquor in the reservoir well. This microseeding protocol led to the growth of single rectangular-shaped crystals within 48 h in a condition consisting of 0.1 *M* MMT (dl-­malic acid, MES and Tris base in a 1:2:2 molar ratio) buffer pH 6, 25%(*w*/*v*) PEG 1500. The crystals were cryoprotected by a direct and brief transfer into cryobuffer [16%(*v*/*v*) glycerol, 16%(*v*/*v*) ethylene glycol, 18%(*w*/*v*) sucrose, 4%(*w*/*v*) glucose] before being flash-cooled in liquid nitrogen.

### 2.3. Data collection and processing

A complete data set was collected from a single crystal on beamline I02 at the Diamond Light Source synchrotron-radiation facility. Automated data processing was performed by *xia*2 (Winter, 2010 ▶). The crystals belonged to space group *P*3_1_, with unit-cell parameters *a* = 69.99, *b* = 69.99, *c* = 91.68 Å, and contained two molecules in the asymmetric unit.

### 2.4. Structure determination

The structure was solved by molecular replacement with *Phaser *(McCoy *et al.*, 2007 ▶) using the structure of a cobalamin-binding protein from *Moorella thermoacetica*, a protein homologous to the C-terminus of CobDH (PDB entry 1y80; Southeast Collaboratory for Structural Genomics, unpublished work; Zhou *et al.*, 2005 ▶; Das *et al.*, 2007 ▶), as a search model and was refined with *REFMAC* (Murshudov *et al.*, 2011 ▶; Winn *et al.*, 2011 ▶). The missing 86 residues of the N-terminal domain could be clearly identified in the electron-density maps which were built with several cycles of automated model building with *Buccaneer* (Cowtan, 2006 ▶). The cobalamin cofactor was manually built into the structure afterwards, followed by iterative rounds of manual building in *Coot* (Emsley *et al.*, 2010 ▶) and crystallographic refinement in *REFMAC* (Murshudov *et al.*, 2011 ▶). An updated B12.cif parameter file was used for refinement of the cobalamin cofactor because the current definition file in *REFMAC* contains an incorrect double bond between C19 and N24 of the cobalamin corrin ring instead of a single bond.

The final quality of the structure in complex with cobalamin was validated using *PROCHECK* (Laskowski *et al.*, 1993 ▶) and *MolProbity* (Chen *et al.*, 2010 ▶). Data-processing and refinement statistics are given in Table 1 ▶. Crystallographic figures were generated using *PyMOL* (http://www.pymol.org).

### 2.5. UV–Vis spectroscopy

UV–Vis absorbance spectra were recorded using a Cary UV–Vis spectrophotometer. All spectra were baseline-corrected with the respective buffer solution. Spectra of the protein reconstituted with methylcobalamin were recorded in the fully oxidized cob(III)alamin form (under aerobic conditions). Anaerobic spectra were recorded within an anaerobic glove box (Belle Technology, O_2_ < 5 p.p.m.) and the bound cobalamin was reduced with titanium(III) citrate. Titanium(III) citrate was prepared in an anaerobic glove box by the addition of sodium citrate to a stirred anaerobic 12% solution of titanium(III) chloride (Sigma) at room temperature (Goulding *et al.*, 1997 ▶). This solution was neutralized by the addition of saturated sodium bicarbonate, yielding a final titanium(III) citrate stock solution of approximately 50 m*M*. CobDH (1 ml at 50 µ*M*) was stepwise reduced by the addition of 0.5 µl aliquots of titanium(III) citrate. The low redox potential of titanium(III) citrate (∼−500 mV) was sufficient to reduce cobalamin to the +1 cobalt oxidation state. The percentages of cobalamin in the different oxidation states were estimated using the extinction coefficients ∊ = 26 000 *M* ^−1^ cm^−1^ for cob(I)alamin at 388 nm, ∊ = 9470 *M* ^−1^ cm^−1^ for cob(II)alamin at 477 nm and ∊ = 9100 *M* ^−1^ cm^−1^ for cob(III)alamin at 540 nm (Goulding *et al.*, 1997 ▶) and a CobDH protein concentration of 50 µ*M*.

### 2.6. EPR spectroscopy

The EPR samples were prepared in the same manner as the UV–Vis samples. Protein concentrations were adjusted to ∼2 mg ml^−1^ (80 µ*M*). Protein samples were reduced with 5 m*M* sodium dithionite in order to obtain the paramagnetic cob(II)alamin species. Samples were transferred aerobically into 4 mm Suprasil quartz EPR tubes (Wilmad), directly frozen and stored in liquid nitrogen until measured. EPR spectra were recorded at 12 and 30 K using a Bruker ELEXSYS E500/E580 EPR spectrometer (Bruker GmbH) fitted with an Oxford Instruments ESR900 helium-flow cryostat coupled to an ITC 503 controller from the same manufacturer. The microwave power was 0.5 mW, the modulation frequency was 100 kHz and the modulation amplitude was 5 G. The *g* values given were calculated using the *Xepr* software package supplied with the instrument.

## 3. Results and discussion

### 3.1. Crystal structure of CobDH

**Figure 1:**
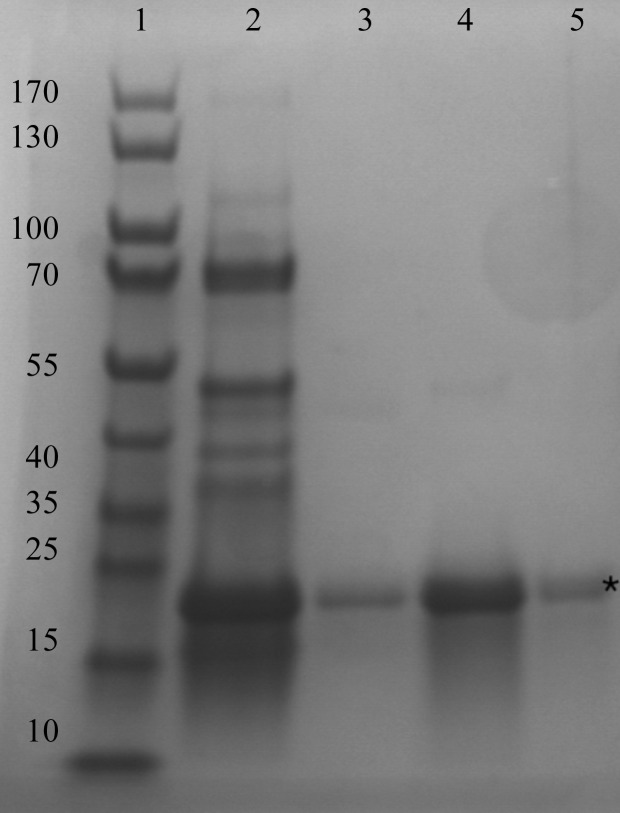
SDS–PAGE of purified CobDH, which has an apparent molecular mass of 22.5 kDa, in line with its calculated molecular mass. Lane 1, marker (labelled in kDa); lane 2, after Ni–NTA; lanes 3–5, after gel filtration. Purified CobDH is indicated by an asterisk. Protein bands were stained with InstantBlue dye (Expedeon).

**Figure 2:**
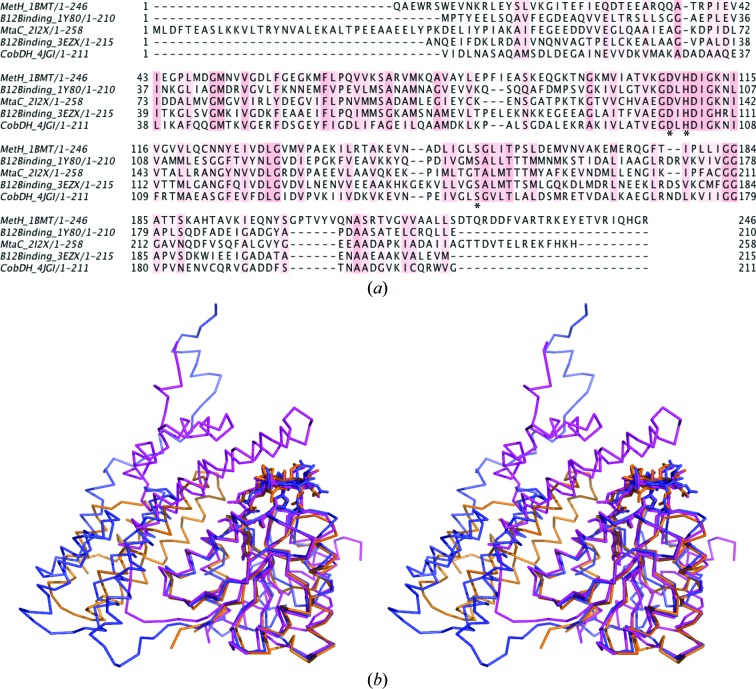
Primary-sequence alignment and superposition of CobDH and homologous proteins for which crystal structures have been solved. (*a*) Multiple sequence alignment of all deposited proteins that bind methylcobalamin in the base-off/His-on form. The histidine side chain from the conserved cobalamin-binding motif D*x*H*xx*G forms a coordination bond with the central Co atom in all cases. Conserved residues of CobDH (Asp100, His102 and Ser147) are marked with asterisks. The alignment was performed using *ClustalX* (Larkin *et al.*, 2007 ▶). (*b*) Stereoview of the superposition of CobDH (orange) with MetH (PDB entry 1bmt; magenta; r.m.s. = 0.774 Å; Drennan *et al.*, 1994 ▶) and MtaC (PDB entry 2i2x; blue; r.m.s. = 0.772; Hagemeier *et al.*, 2006 ▶). Protein backbone traces are shown in ribbon representation and the cobalamins and bound histidines are shown as sticks. In MetH the N-­terminal helix-bundle domain covers the cobalamin from the top, whereas in CobDH and MtaC it is oriented parallel to the cobalamin-binding domain.

Recombinant production of soluble CobDH was successfully achieved in *E. coli* as described in §[Sec sec2]2. The protein consists of 212 residues on a single polypeptide chain, corresponding to a calculated molecular weight of ∼22.5 kDa, which is in line with SDS–PAGE analysis of purified CobDH (Fig. 1 ▶). Given the similarity of CobDH to other cobalamin-binding proteins and the presence of the cobalamin-binding motif D*x*H*xx*G (residues 100–105; Fig. 2 ▶ *a*), it was anticipated that CobDH would bind cobalamin. CobDH was reconstituted during the purification process with exogenous methyl­cobalamin, as the heterologous *E. coli* host only synthesizes methylcobalamin when supplied with cobinamide (Lawrence & Roth, 1995 ▶). Reconstitution was verified by the bright pink colour of purified CobDH, which is a characteristic absorbance feature of cob(III)alamin, indicating successful *in vitro* reconstitution of the protein with methylcobalamin.

Microseeding with small CobDH crystals obtained from initial screening led to the growth of large rectangular crystals which diffracted to a resolution of 1.5 Å. A complete data set was recorded from a single crystal on beamline I02 at the Diamond Light Source. The data were indexed in space group *P*3_1_, with unit-cell parameters *a* = *b* = 69.99, *c* = 91.68 Å. Data-collection and refinement statistics are shown in Table 1 ▶. The structure was solved by molecular replacement using the crystal structure of a cobalamin-binding protein from *M. thermoacetica* (PDB entry 1y80) as a search model. Two CobDH monomers were found in the asymmetric unit.

**Figure 3:**
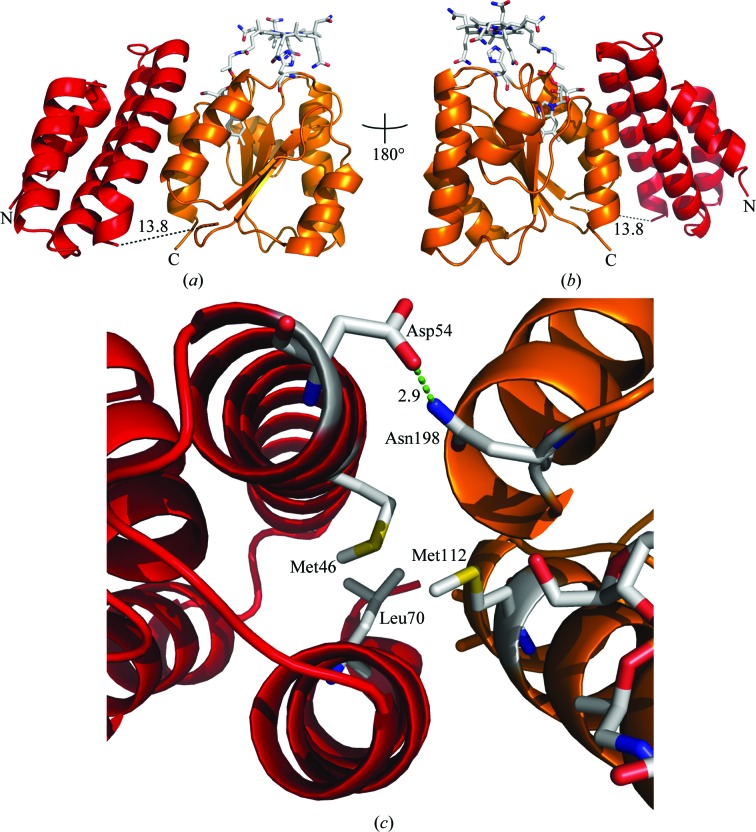
Overall structure of CobDH (cartoon representation) with bound methylcobalamin. (*a*, *b*) The N-­terminal helix-bundle domain is shown in red and the C-terminal Rossmann-fold domain is shown in orange. The central Co atom of the cofactor (shown as sticks; C atoms coloured grey) is coordinated to His102 as the lower axial ligand. The black dotted line connects the domains to each other and represents the missing linker region for which no electron density was observed (residues 83–89). (*c*) Domain interactions between the helix-bundle domain (red) and the cobalamin-binding domain (orange). Asp54 and Asn198 form a hydrogen bond; Met46, Leu70 and Met112 are involved in hydrophobic interactions.

**Figure 4:**
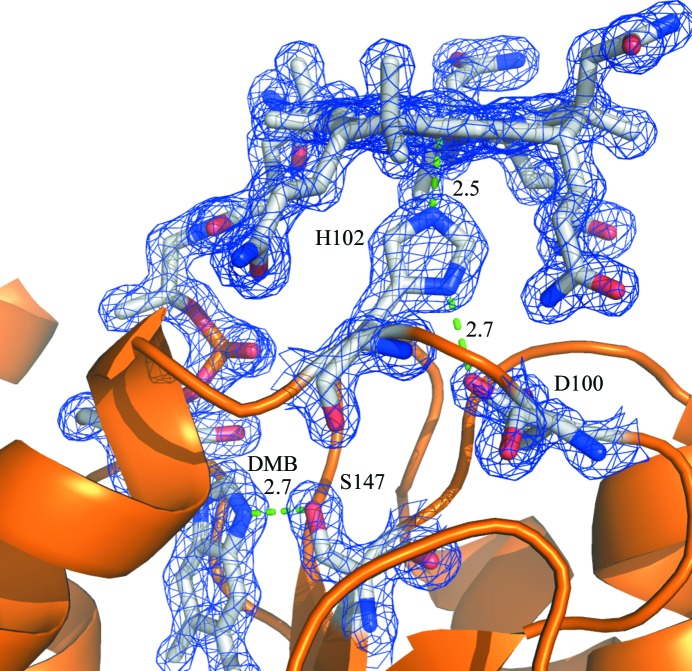
Cobalamin-binding site in CobDH. The cobalamin and the conserved residues Asp100, His102 and Ser147 are shown as sticks (C atoms coloured grey) and are labelled using the single-letter amino-acid codes for clarity. Asp100 hydrogen-bonds to His102 (2.7 Å distance), which in turn is coordinated to the Co atom of the cofactor from the lower axial site (2.5 Å distance). The side chain of Ser147 forms a hydrogen bond to an N atom of the dimethylbenzimidazole (DMB) of the cobalamin (2.7 Å distance). The interactions described are indicated with green dotted lines. The blue mesh corresponds to a 2*F*_o_ − *F*_c_ map with the cobalamin and the key residues omitted from map calculations. The map was contoured at 2.0σ.

The overall high-resolution structure of CobDH (Fig. 3 ▶) reveals two domains that are separated by a flexible linker. The N-terminal domain (residues 1–82) folds into an antiparallel four-helix bundle. The C-terminal domain (residues 90–212) resembles a typical Rossmann fold with a central five-stranded parallel β-sheet surrounded by six α-helices that forms the binding site for the methylcobalamin. The corrin ring and the central Co atom are presented on the surface of the protein, while the dimethylbenzimidazole (DMB) base protrudes deep into the central β-sheet region. The planar corrin ring is oriented perpendicular with respect to the parallel β-sheet core. His102 forms a coordination bond towards the Co atom from the lower axial site, which results in a base-off/His-on cobalamin-binding form. No electron density has been observed at the upper axial ligand site from the Co atom. This finding leads to the conclusion that the methyl group in the crystals has been cleaved off by photoreduction during crystallization, cryoprotection or X-ray exposure, a process that has also been described for other crystal structures of methylcobalamin-dependent enzymes (Hagemeier *et al.*, 2006 ▶; Datta *et al.*, 2008 ▶), resulting in the formation of cob(II)alamin. It is hence assumed that the oxidation state of cobalamin in the described CobDH crystals is +2. His102 is part of a loop that connects the most N-terminal β-­strand of the Rossmann-fold domain to the first α-helix of the domain. Structural alignments show that the entire loop is highly conserved, confirming its key role in cobalamin binding (Fig. 2 ▶). His102 NE2 forms a coordination bond with the central Co atom of cobalamin at a distance of 2.5 Å. ND1 from the same histidine side chain hydrogen-bonds to the side-chain O atom of the conserved Asp100. The side chain of the conserved residue Ser147 forms a hydrogen bond to an N atom of the DMB from the cofactor (2.7 Å distance), as shown in Fig. 4 ▶.

No electron density was observed corresponding to the long linker region which connects the two domains (residues 83–89), suggesting a high degree of flexibility within the linker. This indicates that both domains can undergo structural rearrangements with respect to each other as part of the catalytic cycle of CobDH. However, there are notable interactions between the domains, suggesting that the observed conformation in the crystal structure is of physiological relevance. This is further supported by the fact that the crystal packing of CobDH does not influence the position of the N-terminal four-helix bundle domain. The average *B* factors of the four-helix bundle domain and the Rossmann-fold domain are 29.8 and 24.04 Å^2^, respectively, suggesting that the four-helix bundle domain is less restrained by the lattice. The interactions between both domains are centred around the side chains of Asp54 and Asn198, which form a hydrogen bond (2.9 Å distance), and hydrophobic interactions between the domains that are formed between the side chains of Met46, Leu70, Ile108 and Met112 (Fig. 3 ▶ *c*).

### 3.2. Spectroscopic analysis

**Figure 5:**
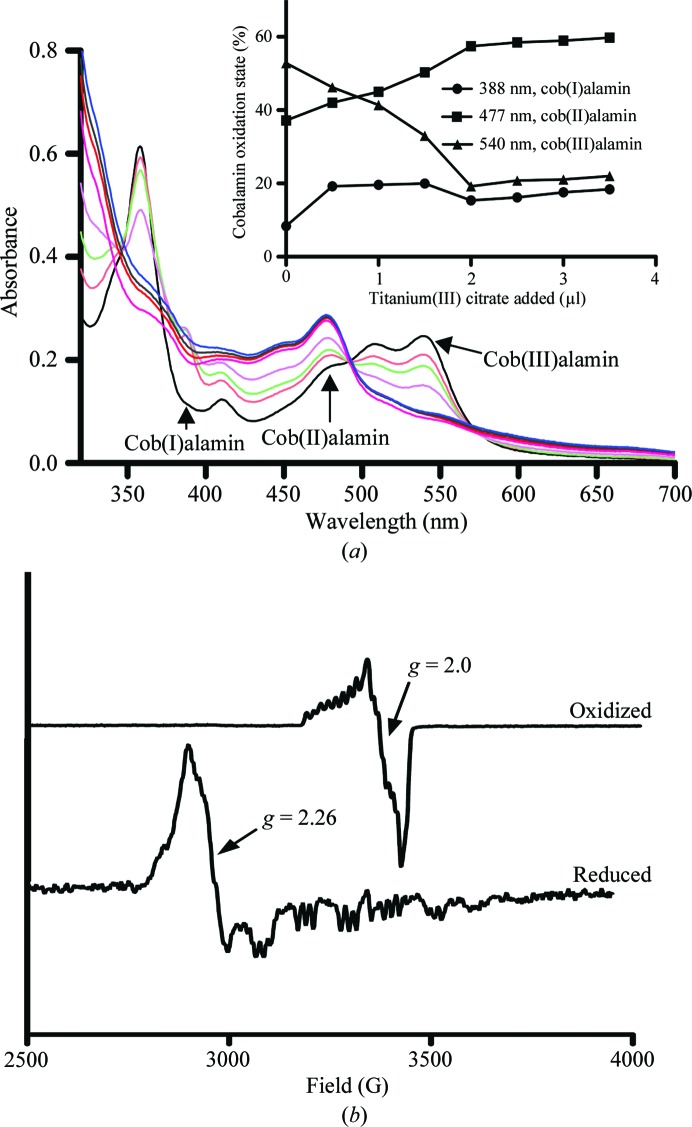
UV–Vis and EPR analyses of CobDH. (*a*) Anaerobic titration of oxidized CobDH with titanium(III) citrate as a reductant. The absorbance maximum of cob(III)alamin at 540 nm decreases with increasing amounts of added reductant; the absorbance of cob(II)alamin (474 nm) and cob(I)alamin (388 nm) increase correspondingly. The inset shows a plot of the percentage of CobDH in each of the three possible oxidation states of its cofactor during titration with titanium(III) citrate. The respective oxidation-state percentages were calculated as described in §[Sec sec2]2. The cob(III)alamin percentage in CobDH decreases from approximately 53 to 22%, whereas the percentages of cob(II)alamin and cob(I)alamin increase from 37 to 60% and from 8 to 18%, respectively. (*b*) The oxidized EPR sample refers to CobDH as purified. The cob(III)alamin–superoxide complex signal has a *g* value of 2.0 (magnetic field of 3400 G; upper spectra). After sample reduction with 5 m*M* sodium dithionite the spectrum shows the presence of the base-off/His-on form with a main feature at *g* = 2.26 (magnetic field of 2900 G; lower spectra). Also present is hyperfine splitting of the signal, which arises from the interaction between the cob(II)alamin and its β-­axial coordinated N atom from the His102 side chain.

The UV–Vis absorbance spectrum of reconstituted CobDH (Fig. 5 ▶ *a*) in the oxidized state revealed characteristic features of methyl(III)cobalamin (540 nm maximum), in addition to peaks at 520, 410 and 350 nm that have previously been observed for hydroxo(III)cobalamin (Jarrett *et al.*, 1997 ▶). These observations indicate that the coordination bond between the cobalamin Co atom and the upper axial methyl ligand is cleaved to a large extent, leading to the formation of hydroxo(III)cobalmin or aquacobalamin. This cleavage can be attributed to the exposure of the sample to light and oxygen during the protein-purification steps. Under reducing and anaerobic conditions [the addition of titanium(III) citrate in an anaerobic glove box], distinct changes occur in the spectra. The features at 540, 520, 410 and 350 nm disappear and are replaced by cob(II)alamin-specific and cob(I)alamin-specific peaks at 477 and 388 nm. Quantification of the peaks at 540, 477 and 388 nm was performed in order to estimate the percentages of cobalamin-bound protein in the +3, +2 and +1 oxidation states, respectively. The inset in Fig. 5 ▶(*a*) shows a plot of these percentages *versus* the amount of titanium(III) citrate added, revealing that the percentage of cob(III)alamin decreases from approximately 53 to 22%, whereas the percentages of cob(II)alamin and cob(I)alamin increase from 37 to 60% and from 8 to 18%, respectively. The calculated percentages are in good agreement with the assumption of complete reconstitution of CobDH with cobalamin.

Similar absorbance changes have been observed for *E. coli* MetH during methyl transfer from methyltetrahydrofolate (methyl donor) to exogenous cob(I)alamin and from methyl(III)cobalamin to homocysteine (methyl acceptor; Goulding *et al.*, 1997 ▶). EPR analysis (see Fig. 5 ▶ *b*) was used to further characterize the cofactor-binding mode in CobDH. The EPR spectrum of CobDH in the absence of any reductant (upper spectra in Fig. 5 ▶ *b*, prepared under aerobic conditions) exhibits the characteristic features of a cob(III)alamin–superoxide complex, which gives rise to an EPR signal at *g* = 2.0 owing to the presence of an unpaired electron that is predominantly located on the complexed oxygen molecule (Hoffman *et al.*, 1970 ▶). Upon the addition of a suitable reductant (here sodium dithionite), the Co atom is reduced to the +2 state and exhibits the characteristic features of a base-off/His-on form at *g* = 2.26, which is in agreement with the position of His102 observed in the crystal structure. Our spectroscopic data indicate that the CobDH-bound cobalamin is able to cycle between different configurations: cob(I)alamin is tetracoordinated (lacking both the lower and the upper axial ligands), the paramagnetic cob(II)­alamin species is pentacoordinated by His102 from the lower axial site to the central cobalt and cob(III)alamin is usually diamagnetic, but our EPR spectra suggest the presence of a paramagnetic cob(III)alamin–superoxide complex. To our knowledge, protein-bound cob(III)alamin–superoxide complexes have only previously been identified for *E. coli* MetH (Frasca *et al.*, 1988 ▶).

### 3.3. Comparison of CobDH with other methyltransferases

Fig. 2 ▶(*a*) shows a multiple sequence alignment between CobDH (PDB entry 1y80; the molecular-replacement model used) and other close homologues in the PDB. All structures were solved bound to methylcobalamin, except for the protein from *Methanosarcina barkeri* (PDB entry 3ezx; R. Jain, B. Hao, J. A. Soares, L. Zhang, K. Green-Church, X. Li, J. A. Krzycki & M. K. Chan, unpublished work), which was solved bound to hydroxocobalamin. The sequence alignment reveals that only the cobalamin-binding region shows high conservation (Matthews *et al.*, 2008 ▶). The cobalamin-binding motif D*x*H*xx*G is the only common primary-sequence motif that is conserved between the methyltransferases and isomerases, while it is absent in the reductive dehalogenases.

All aligned structures contain two domains. The structurally conserved C-terminal cobalamin-binding domain is responsible for the correct positioning of the cobalamin corrin ring on the surface of the Rossmann-fold domain. Hence, it is easily accessible to other protein modules that are required for catalysis, for example methyl-donor and/or methyl-acceptor domains and their respective ligands (*e.g.* 5-methyltetrahydrofolate, methanol, homocysteine, coenzyme M *etc.*).

In contrast, the function of the second domain, the N-­terminal four-helix bundle, is less well understood. The high flexibility of the linker domain between the domains could promote the structural rearrangement of both domains during the catalytic cycle with respect to each other. The orientation of the N-terminal four-helix bundle domain with respect to the C-terminal domain is indeed variable across the family. This N-terminal domain can either be oriented parallel to the cobalamin-binding domain or it can shield the cofactor upper axial site (as is the case in MetH; Fig. 2 ▶ *b*) from the environment, thus preventing putative interactions between solvent molecules and the attached methyl group (Drennan *et al.*, 1994 ▶). It has been shown crystallographically for MetH that the N-terminal four-helix bundle domain moves 26 Å away from the cobalamin in order to allow interactions between cobalamin and the activation domain (AdoMet binding), which is the most C-terminal domain of MetH (Bandarian *et al.*, 2002 ▶).

MetH is a large multidomain protein with four distinct modules, whereas CobDH and MtaC only comprise the cobalamin-binding domain, which might further explain the different positions of the four-helix bundle domain with respect to the cobalamin cofactor. For CobDH and MtaC this domain is parallel to the cobalamin-binding domain and does not interact with the cofactor (Fig. 2 ▶ *b*). Furthermore, in the case of the methanol MtaABC system it has been proposed that the helix-bundle domain of MtaC is permanently anchored to the methanol-binding protein MtaB, allowing the cobalamin-binding domain of MtaC to rotate and bind to either the methanol-binding region of MtaB or the methyl-donor region of MtaA, which is the third protein involved in the methanol-cleavage reaction (Hagemeier *et al.*, 2006 ▶; Hoeppner *et al.*, 2012 ▶).

It would be interesting to find out whether such major movements of the helix-bundle domain also occur at some stage along the catalytic reaction coordinate or during reactivation processes in the multiprotein *O*-demethylase system. Bioinformatic studies propose that CobDH is part of a multi-component *O*-demethylase enzyme system (Studenik *et al.*, 2012 ▶). The analysis indicates that in the same operon as CobDH (gene *Dhaf0720*), the genes *Dhaf0721* and *Dhaf0722* code for methyl-donor (substrate-binding) and methyl-acceptor (tetrahydrofolate-binding) domains, respectively. This scenario promotes a multi-component arrangement of the *O*-demethylase system similar to the methanol-cobalamin methyltransferase complex (Hagemeier *et al.*, 2006 ▶) and would hence explain the parallel orientation of the four-helix bundle to the cobalamin-binding domain in CobDH.

## 4. Conclusions

We have identified and isolated a previously unknown cobalamin-binding protein (CobDH) from *D. hafniense* and solved its crystal structure. The structure reveals the characteristic base-off/His-on cobalamin-binding site with the cobalamin coordinated to His102 as part of a conserved sequence motif observed in other cobalamin-dependent methyltransferases and implies that CobDH likewise catalyses the transfer of a methyl group. EPR and UV–Vis spectroscopic analyses of CobDH confirmed the cobalamin binding and revealed that the CobDH-bound cobalamin cofactor is able to cycle between the cobalt(I), cobalt(II) and cobalt(III) oxidation states. However, as with the other methyltransferases studied, additional proteins or protein domains are needed to bind the substrates and products and present these to the upper axial position of the cobalamin moiety for catalysis. The genomic context of the CobDH gene suggests that it is part of a three-component *O*-­demethylase enzyme system. *O*-Demethylase activity has been observed in *D. hafniense* strains DCB-2 and PCP-1 and recent studies have established *O*-demethylase activity for a related operon consisting of *Dhaf4610*, *Dhaf4611* and *Dhaf4612* when expressed in *E. coli* (Studenik *et al.*, 2012 ▶). This suggests that organohalide-respiring bacteria not only use cobalamin to support the dechlorination of halogenated compounds, but also make extensive use of this cofactor in catalysis of demethylation reactions, leading to further mineralization. With respect to bioremediation strategies and a recent study (Villemur, 2013 ▶), it would be especially interesting to biochemically confirm the presence of shared substrates in *D. hafniense* (or any other organohalide-respiring bacteria) between *O*-demethylases and reductive dehalogenases. We hence intend to investigate the functional relationships between the *Dhaf0720–0722* gene products on both a biochemical and a structural level to further explore the versatility of cobalamin-catalysed enzymatic reactions in organohalide-respiring bacteria.

## Supplementary Material

PDB reference: CobDH, 4jgi

## Figures and Tables

**Table 1:** Crystallographic data and refinement statistics Values in parentheses are for the highest resolution shell.

Data collection	
X-ray source	Beamline I02, Diamond
Wavelength (Å)	0.946
Space group	*P*3_1_
Temperature (K)	100
Unit-cell parameters (Å)	*a* = *b* = 69.99, *c* = 91.68
Resolution range (Å)	32.7–1.5 (1.54–1.50)
Observed reflections	79674 (3997)
Multiplicity	9.3 (8.6)
Completeness (%)	99.0 (97.3)
*R*_merge_ † (%)	10 (85)
*R*_meas_ ‡ (%)	11.4 (106)
CC_1/2_ (%)	99.7 (61.1)
〈*I*/σ(*I*)〉	10.5 (3.5)
Wilson *B* factor (Å^2^)	18.50
No. of protein molecules in asymmetric unit	2
Matthews coefficient *V*_M_ (Å^3^ Da^−1^)	2.51
Solvent content (%)	50.6
Refinement	
Resolution (Å)	32.7–1.5 (1.54–1.50)
*R*_work_/*R*_free_ § (%)	14.7/19.4
No. of protein atoms	3247
No. of water atoms	448
Overall *B* factor (Å^2^)	26
Ramachandran statistics	
Favoured (%)	98.26
Allowed (%)	1.49
Outliers (%)	0.25
R.m.s.d., bonds (Å)	0.021
R.m.s.d., angles (°)	2.985
PDB code	4jgi

† *R*_merge_ = 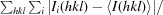 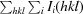.

‡ *R*_meas_ = 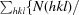 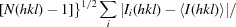 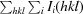, where *I*_*i*_(*hkl*) are the observed intensities, 〈*I*(*hkl*)〉 is the average intensity and *N*(*hkl*) is the multiplicity of reflection *hkl*.

§ *R*_work_ = 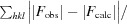 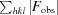. *R*_free_ is the cross-validation *R* factor for the test set (5%) of reflections omitted from model refinement.
